# Progression of Liver Fibrosis in HIV/HCV Co-Infection: A Comparison between Non-Invasive Assessment Methods and Liver Biopsy

**DOI:** 10.1371/journal.pone.0138838

**Published:** 2015-09-29

**Authors:** Patrick Schmid, Andrea Bregenzer, Milo Huber, Andri Rauch, Wolfram Jochum, Beat Müllhaupt, Pietro Vernazza, Milos Opravil, Rainer Weber, Swiss HIV Cohort Study

**Affiliations:** 1 Division of Infectious Diseases and Hospital Epidemiology, Cantonal Hospital St. Gallen, St. Gallen, Switzerland; 2 Division of Infectious Diseases and Hospital Epidemiology, University Hospital Zurich, University of Zurich, Zurich, Switzerland; 3 Division of Infectious Diseases and Hospital Epidemiology, University Hospital Berne, Berne, Switzerland; 4 Institute of Pathology, Cantonal Hospital St. Gallen, St. Gallen, Switzerland; 5 Division of Hepatology, University Hospital Zurich, Zurich, Switzerland; SAINT LOUIS UNIVERSITY, UNITED STATES

## Abstract

**Objectives:**

To evaluate the diagnostic performance of seven non-invasive tests (NITs) of liver fibrosis and to assess fibrosis progression over time in HIV/HCV co-infected patients.

**Methods:**

Transient elastography (TE) and six blood tests were compared to histopathological fibrosis stage (METAVIR). Participants were followed over three years with NITs at yearly intervals.

**Results:**

Area under the receiver operating characteristic curve (AUROC) for significant fibrosis (> = F2) in 105 participants was highest for TE (0.85), followed by FIB-4 (0.77), ELF-Test (0.77), APRI (0.76), Fibrotest (0.75), hyaluronic acid (0.70), and Hepascore (0.68). AUROC for cirrhosis (F4) was 0.97 for TE followed by FIB-4 (0.91), APRI (0.89), Fibrotest (0.84), Hepascore (0.82), ELF-Test (0.82), and hyaluronic acid (0.79). A three year follow-up was completed by 87 participants, all on antiretroviral therapy and in 20 patients who completed HCV treatment (9 with sustained virologic response). TE, APRI and Fibrotest did not significantly change during follow-up. There was weak evidence for an increase of FIB-4 (mean increase: 0.22, p = 0.07). 42 participants had a second liver biopsy: Among 38 participants with F0-F3 at baseline, 10 were progessors (1-stage increase in fibrosis, 8 participants; 2-stage, 1; 3-stage, 1). Among progressors, mean increase in TE was 3.35 kPa, in APRI 0.36, and in FIB-4 0.75. Fibrotest results did not change over 3 years.

**Conclusion:**

TE was the best NIT for liver fibrosis staging in HIV/HCV co-infected patients. APRI-Score, FIB-4 Index, Fibrotest, and ELF-Test were less reliable. Routinely available APRI and FIB-4 performed as good as more expensive tests. NITs did not change significantly during a follow-up of three years, suggesting slow liver disease progression in a majority of HIV/HCV co-infected persons on antiretroviral therapy.

## Background

HIV co-infection accelerated progression of liver fibrosis in hepatitis C virus (HCV)-infected patients before potent antiretroviral therapy (ART) became available [1]. Whether HIV infection is still a relevant risk factor for liver disease progression under suppressive ART is controversial [2–5]. Reliable staging of liver fibrosis is essential for clinical decision making. Liver biopsy has been traditionally considered as gold standard to evaluate liver fibrosis. However, this procedure has several drawbacks such as pain and bleeding complications, and it can also lead to inaccurate staging due to sampling error and variability in the interpretation of biopsies. In the last decade various non-invasive tests (NITs) have been introduced to stage liver fibrosis of which measurement of liver stiffness by transient elastography (TE) using FibroScan® is the most widely accepted method. This device-dependent technique is mainly limited to larger centres, and sometimes it cannot be applied in obese patients or persons with narrow intercostal spaces. Blood serum markers—singly or in combination—are alternative non-invasive tests that allow a more widespread use and are cheaper. Serum markers as routine biochemical parameters (indirect markers) and markers of hepatic matrix metabolism (direct markers) have been evaluated mainly in HCV-mono-infected patients [6,7]. Fibrotest®, a panel of 5 serum values, is the best validated marker. In HIV/HCV co-infected patients head-to-head comparison of NITs is still limited [8,9].

Until recently, HCV-treatment was associated with poor rates of sustained virological response (SVR), especially in patients with HCV/HIV co-infection. Improved treatments with cure rates >90% are now available in some countries. Because of the high costs of these new HCV drugs, access to treatment is limited to patients with severe fibrosis or cirrhosis in most countries. Therefore, assessment of liver-fibrosis as a key parameter to estimate prognosis will still be required for the indication of antiviral treatment in the coming years.

The aim of this study was to compare the diagnostic accuracy of liver fibrosis staging by TE and six serum biochemical markers in HIV-HCV-co-infected patients. Furthermore, situations in which liver biopsies might safely be replaced by non-invasive tests should be determined. Another goal was to assess changes in liver fibrosis over time. When the study was planned, European guidelines recommended repeated liver biopsies every 3 to 5 years in HIV/HCV co-infected patients without treatment-induced sustained response. Therefore the cohort was longitudinally followed over 3 years.

## Methods

Ethics Committee of St. Gallen, Zurich and Bern have approved this study. All participants have signed an informed consent.

### Study Population

This study was conducted as a nested project of the Swiss HIV Cohort Study (SHCS; www.shcs.ch). HIV positive participants with chronic HCV infection (detectable HCV-RNA by PCR for at least six months) were included, if they agreed with a liver biopsy. The sample size of 100 patients was calculated to determine the different receiver operating characteristic (ROC) curves (as shown in [10] on 130 patients). The computation of the sample size targeted primarily to achieve a good sensitivity for the diagnosis of severe fibrosis. According to the values given by Castera et al. [11], the sensitivity was 73% to diagnose a METAVIR fibrosis stage ≥3. To achieve a 95% confidence interval of 63 to 83% for the true sensitivity of 73%, the necessary number of patients was 76 [12]; Java Applets for Power and Sample Size, http://www.stat.uiowa.edu/~rlenth/Power]. The targeted number of 100 recruited patients included a compensation for missing data or withdrawals.

### Clinical Assessment

All patients had semi-annual study visits as previously described [13]. Reasons for death or drop out were recorded. Liver related morbidities were evaluated with a case-report form.

### Histopathological assessment of liver biopsies (LB)

LB was performed at baseline and three years later. Sections stained with haematoxylin and eosin or CAB were used for histopathologic evaluation using the degree of inflammation, steatosis, and fibrosis as morphological endpoints. Inflammation and fibrosis were scored according to the METAVIR system [14]. The percentage of hepatocytes with fat droplets was estimated to evaluate the degree of liver steatosis. All histopathologic assessments were performed centrally by an experienced pathologist (WJ) who was not aware of the results of the non-invasive liver fibrosis tests and the clinical course of the patients.

### Non-invasive assessment of liver fibrosis

#### Transient elastography (TE)

TE is a painless, ultrasound-based method to measure the mean stiffness of hepatic tissue. For TE, the FibroScan® instrument (Echosens, Paris, France) was used [15]. TE was performed at baseline and yearly intervals.

#### Blood-based assessment of liver fibrosis

Blood samples were obtained at baseline and longitudinally at yearly intervals. Blood was collected in serum separator tubes, centrifuged at 3000 rpm for 10 min, and plasma stored at -80°C. From baseline-samples six non-invasive markers of liver fibrosis were determined. An overview of the components of the different blood markers is given in [Table 1]. Standard cut-offs in the literature are summarized in [Table 2].

**Table 1:** Blood markers for non-invasive diagnosis of liver fibrosis.

TEST	DESCRIPTION
Indirect markers	
APRI	AST, platelets
FIB-4	AST, ALT, platelets, age
Fibrotest	alpha-2-macroglobulin, haptoglobin, apolipoprotein A1, GGT, total bilirubin
Direct markers (hepatic matrix metabolism)	
HYA	hyaluronic acid
Hepascore	alpha-2-macroglobulin, hyaluronic acid, GGT, total bilirubin, age, sex
ELF	hyaluronic acid, type III procollagen, TIMP1, age

**Table 2:** Standard cut-off values of non-invasive tests of liver fibrosis in the literature.

TEST	Absence of significant fibrosis	Presence of significant fibrosis	Absence of cirrhosis	Presence of cirrhosis	Ref.
TE (kPa)	<7.0	> = 7.0	< 10.0	> = 12.5	[22,29]
APRI	< 0.5	> = 1.5	< 1.0	> = 2.0	[16,29,8]
FIB-4	n.a.	n.a.	< 1.45 (<F3)	> = 3.25 (> = F3)	[17]
Fibrotest	< 0.32	> 0.48	< 0.59	> = 0.75	[29,8]
Hyaluronic acid (μg/l)	<16	>121	< 50–60	> 110–237	[34–36]
Hepascore	< 0.5	> = 0.5–0.55	< 0.84	> = 0.84	[19,8,37]
ELF	< 7.7	> = 9.8	< 11.3	> = 11.3	[26]

#### APRI-Score

APRI is score derived from routine blood markers which is calculated based on the formula proposed by Wai et al. [16]: [(AST/ULN (upper limit of normal) of AST) x 100] / platelets (10^9^/L). AST = aspartate aminotransferase.

#### FIB-4 Index

FIB-4 was determined using the formula proposed by Sterling et al. [17]: (age x ASAT) / (platelets (10^9^/L) x ALT^1/2^). ALT = alanine aminotransferase.

#### Fibrotest®

Fibrotest is a composite marker, calculated from α2-macroglobulin, haptoglobulin, apolipoprotein A, GGT and total bilirubin [18]. The algorithm of Fibrotest® is under patent and the calculation was done by Biopredictive® (Paris, France). GGT = gamma-glutamyltransferase.

#### Hyaluronic acid

Hyaluronic acid levels were measured using a commercially available enzyme-linked binding protein assay, HA test kit (Corgenix, Colorado, USA), according to the manufacturer’s protocol.

#### Hepascore

Hepascore is a composite marker, calculated from α-2 macroglobulin, hyaluronic acid, GGT and total bilirubin. Hepascore was calculated using the formula proposed by Adams et al. [19]: y = exp [−4.185818 − (0.0249 x age) + (0.7464 x sex) + (1.0039 x α2-macroglobulin) + (0.0302 x hyaluronic acid) + (0.0691 x bilirubin) − (0.0012 x GGT)]

#### Enhanced Liver Fibrosis (ELF®)-Test

ELF-Test is a composite marker of extracellular matrix components (hyaluronic acid, PIIINP and TIMP-1) [20] merchandised by Siemens Medical Solutions Diagnostics® (Tarrytown, USA).

### Data Analyses

#### Baseline evaluation

The diagnostic accuracy of the non-invasive tests was compared to histopathological staging (liver biopsy = gold standard) by ROC analysis. ROC (x-axis: 1-specificity, y-axis: sensitivity) were calculated for significant fibrosis (> = F2-METAVIR stage) and cirrhosis (F4). The diagnostic accuracy was defined by the area under the ROC-curve (AUROC). Sensitivity (proportion of true positives, correctly identified by the new test), specificity (proportion of true negatives correctly identified by the new test), positive predictive value (PPV) (proportion of individuals with a positive test who actually have the disease) and negative predictive value (NPV) (proportion of individuals with a negative test who actually do not have the disease) were determined for various cut-off points. Optimal cut-off values for each test were established where the sum of sensitivity and specificity was maximal.

#### Longitudinal follow-up

All participants with a complete follow-up of 3 years were analysed. A paired t-test (comparing the population mean difference with zero) was used to determine whether there was a significant change in TE, APRI, FIB-4 or Fibrotest between baseline and 3year follow-up. The subgroup of participants with paired liver biopsies was divided into “progressors” (increase of at least one METAVIR-stage between the two biopsies) and “non-progressors” (no change or decrease in fibrosis stage). Mean changes of non-invasive tests were compared between progressors and non-progressors by t-test, and predictors of fibrosis progression were analysed by logistic regression.

Statistical calculations were performed with Stata, Version 12.0 (Stata Corporation).

## Results

### Baseline parameters of study participants

105 HIV/HCV co-infected participants (81 male, 24 female) were enrolled (05/2006 to 12/2010). HCV-genotype (GT) distribution was: GT 1, 56 participants; GT 2, 4; GT 3, 23; GT 4, 21; and GT 1/4, 1. Mean age was 43 years, mean CD4-count 474/μl (7 patients <200/μl). 86 patients (82%) were on ART at baseline. 21 had an Atazanavir-based ART, which can cause elevated total bilirubin. 24 patients had low platelet counts, 5 of them <100 G/l. Seven patients with thrombocytopenia were not on ART.

### Assessment of liver-fibrosis at baseline

#### Histopathological quantification of liver fibrosis (n = 105)

A standard number of biopsy-passes was not foreseen in the protocol. Three in four of biopsies fulfilled the quality criteria proposed by Colloredo et al. [21], including liver specimens greater than 20 mm in length and more than 11 portal tracts). No or mild fibrosis (METAVIR stage F0 or F1) was observed in 67 patients (64%). 20 patients had moderate (F2), 4 patients severe fibrosis (F3), and 14 patients had cirrhosis (F4). Thus, > = F2-prevalence was 36%, and F4-prevalence 13%. Inflammatory activity was absent or mild (METAVIR grade A0 or A1) in 98 patients, moderate (A2) in 6, and severe (A3) in one patient. Hepatic steatosis was observed in 54 patients (19 patients with steatosis of >30% of hepatocytes).

#### Non-invasive tests

Since only 4/105 (4%) patients had severe fibrosis (F3) at baseline, we restricted our validation of the NITs to the detection of significant fibrosis (> = F2) and cirrhosis (F4).

**Fig 1:**
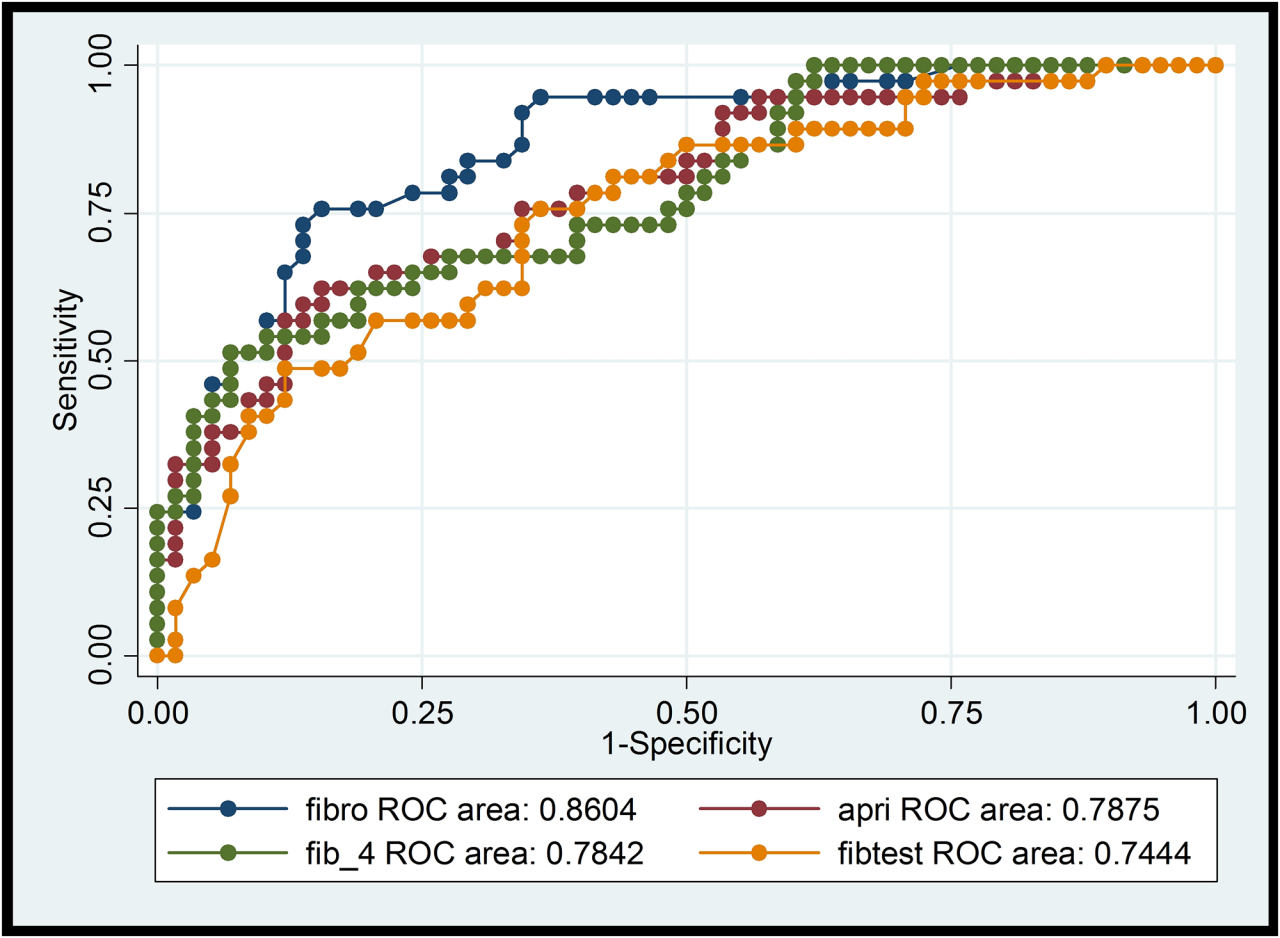
ROCs of transient elastography, APRI, FIB-4, Fibrotest for significant fibrosis (F> = 2). ROC: receiver operating characteristic curve; fibro: transient elastography (TE); fibtest: Fibrotest®.

**Fig 2:**
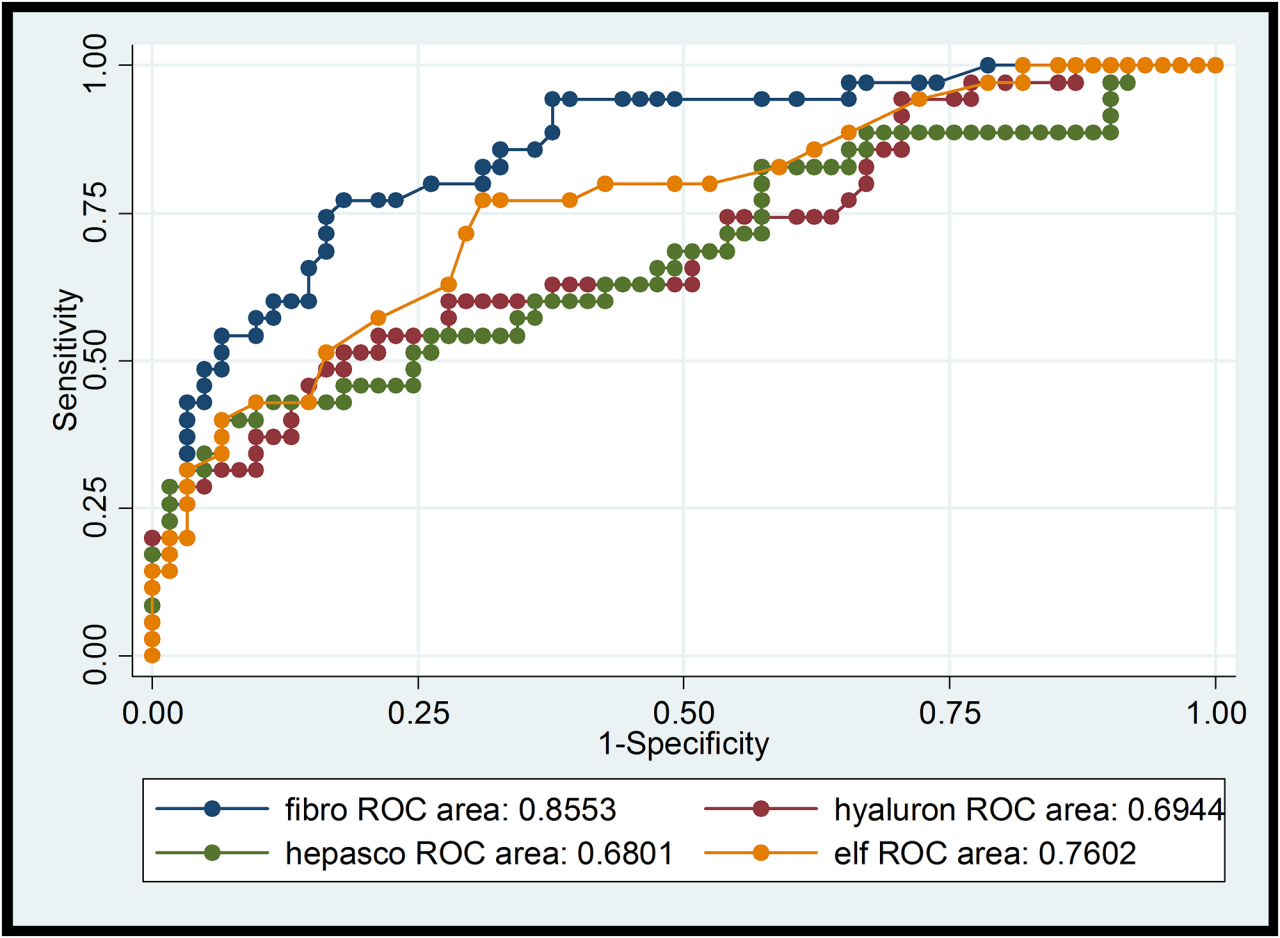
ROCs of transient elastography, hyaluronic acid, Hepascore, ELF-Test for significant fibrosis (F> = 2). ROC: receiver operating characteristic curve; fibro: transient elastography (TE); hyaluron: hyaluronic acid; hepasco: Hepascore, ELF: Enhanced Liver Fibrosis-Test®.

**Fig 3:**
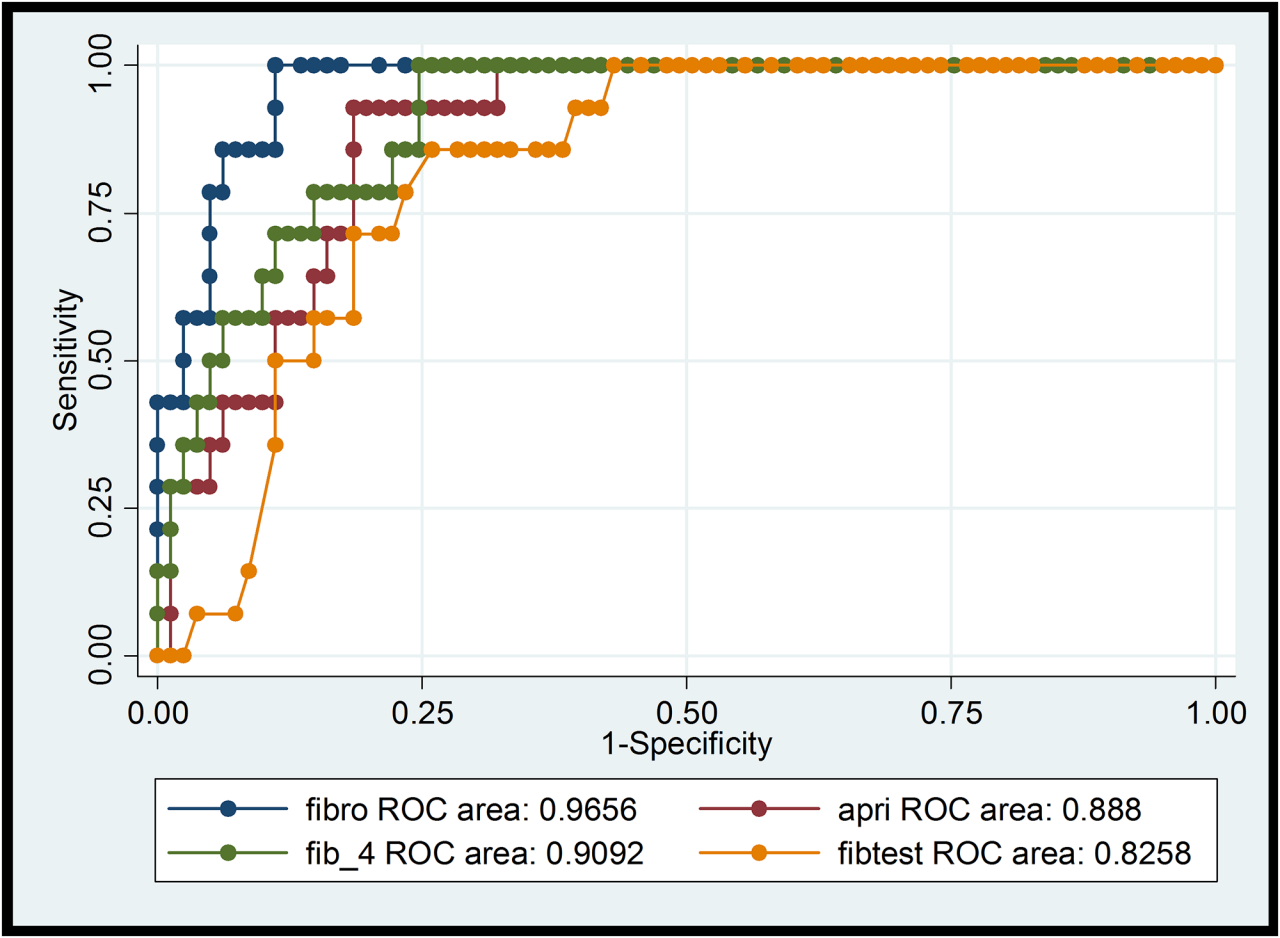
ROCs of transient elastography, APRI, FIB-4, Fibrotest for cirrhosis (F4). ROC: receiver operating characteristic curve; fibro: transient elastography (TE); fibtest: Fibrotest®.

**Fig 4:**
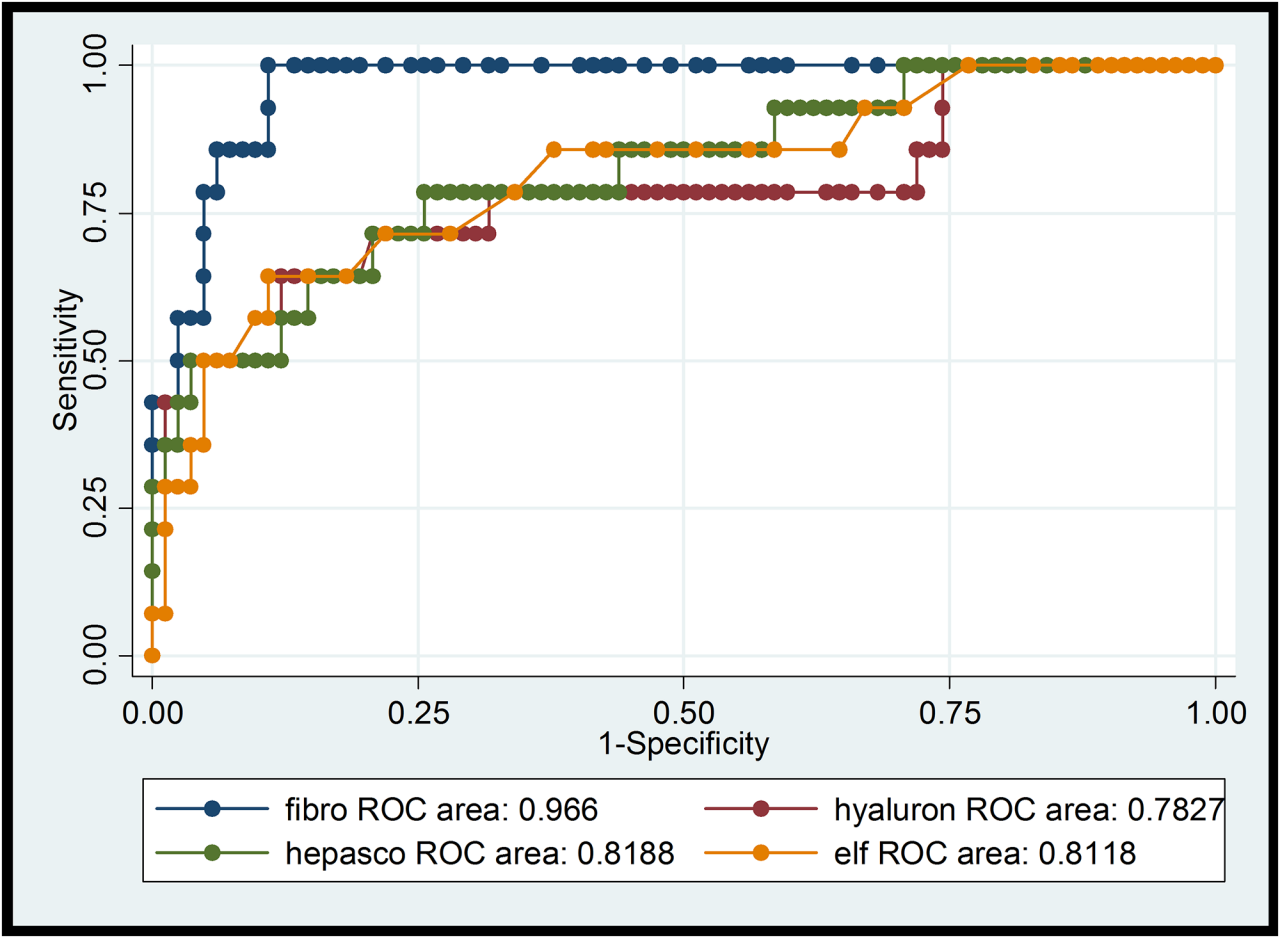
ROCs of transient elastography, hyaluronic acid, Hepascore, ELF-Test for cirrhosis (F4). ROC: receiver operating characteristic curve; fibro: transient elastography (TE); hyaluron: hyaluronic acid; hepasco: Hepascore, ELF: Enhanced Liver Fibrosis-Test®.

Comparison of the different NITs for the diagnosis of significant fibrosis (F≥2) and cirrhosis (F4) is summarized in Tables 3 and 4 including sensitivity and specificity for standard cut-off values as well as for the optimized method defined above (maximized sensitivity+specificity). The respective ROC curves with the AUROC values are shown in Figs 1–4.

**Table 3:** Performance of non-invasive tests for diagnosis of significant fibrosis (> = F2).

TEST	n	≥F2 prevalence in biopsy (%)	Cut-off Value	Cut-off Basis	Sens (%)	Spec (%)	PPV (%)	NPV (%)	Sens+Spec (%)	Correctly classified (%)
**TE**	99	37.4 (37/99)	≥7.2 kPa	Sens + Spec = Max	75.7 (28/37)	82.3 (51/62)	71.8 (28/39)	85.0 (51/60)	158.0	79.8 (79/99)
			≥7.0 kPa	Standard [22]	75.7 (28/37)	79.0 (49/62)	68.3 (28/41)	84.5 (49/58)	154.7	77.8 (77/99)
**APRI**	104	36.5 (38/104)	≥1.1	Sens + Spec = Max	55.3 (21/38)	87.9 (58/66)	72.4 (21/29)	77.3 (58/75)	143.2	76.0 (79/104)
			≥0.5	Standard [16,29,8]	78.9 (30/38)	48.5 (32/66)	46.9 (30/64)	80.0 (32/40)	127.4	59.6 (62/104)
			>1.5	Standard [16,29,8]	36.8 (14/38)	92.4 (61/66)	73.7 (14/19)	71.8 (61/85)	129.2	72.1 (75/104)
**FIB-4**	103	35.9 (37/103)	≥2.63 (?)	Sens + Spec = Max	51.4 (19/37)	92.4 (61/66)	79.2 (19/24)	77.2 (61/79)	143.8	77.7 (80/103)
			≥1.45(<F3)	Standard [17]	73.0 (27/37)	59.1 (39/66)	50.0 (27/54)	79.6 (39/49)	132.1	64.1 (66/103)
**Fibro-test**	101	36.6 (37/101)	≥0.60	Sens + Spec = Max	75.7 (28/37)	64.1 (41/64)	54.9 (28/51)	82.0 (41/50)	139.8	68.3 (69/101)
			>0.1	Standard [29,8]	100 (37/37)	3.1 (2/64)	37.4 (37/99)	100 (2/2)	103.1	38.6 (39/101)
			≥0.32	Standard [29,8]	94.6 (35/37)	28.1 (18/64)	43.2 (35/81)	90.0 (18/20)	122.7	52.5 (53/101)
			>0.48	Standard [29,8]	86.5 (32/37)	48.4 (31/64)	49.2 (32/65)	86.1 (31/36)	134.9	62.4 (63/101)
**Hya**	102	35.3 (36/102)	≥60.1	Sens + Spec = Max	52.8 (19/36)	81.8 (54/66)	61.3 (19/31)	76.1 (54/71)	134.6	71.6 (73/102)
			≥16	Standard [36]	100 (36/36)	4.5 (3/66)	36.4 (36/99)	100 (3/3)	104.5	38.2 (39/102)
			≥59.5	Standard [34]	52.8 (19/36)	81.8 (54/66)	61.3 (19/31)	76.1 (54/71)	134.6	71.6 (73/102)
			>121	Standard [36]	27.8 (10/36)	98.5 (65/66)	90.9 (10/11)	71.4 (65/91)	126.3	73.5 (75/102)
**Hepa-score**	102	35.3 (36/102)	≥0.977 (?)	Sens + Spec = Max	38.9 (14/36)	93.9 (62/66)	77.8 (14/18)	73.8 (62/84)	132.8	74.5 (76/102)
			≥0.5	Standard [19,8]	75.0 (27/36)	43.9 (29/66)	42.2 (27/64)	76.3 (29/38)	118.9	54.9 (56/102)
			≥0.55	Standard [37]	69.4 (25/36)	48.5 (32/66)	42.4 (25/59)	74.4 (32/43)	117.9	55.9 (57/102)
**ELF**	102	34.3 (35/102)	≥9.0	Sens + Spec = Max	77.1 (27/35)	68.7 (46/67)	56.3 (27/48)	85.2 (46/54)	145.8	71.6 (73/102)
			≥7.7	Standard [26]	100 (35/35)	19.4 (13/67)	39.3 (35/89)	100 (13/13)	119.4	47.1 (48/102)
			≥9.8	Standard [26]	40.0 (14/35)	92.5 (62/67)	73.7 (14/19)	74.7 (62/83)	132.5	74.5 (76/102)

TE: transient elastography; Hya: hyaluronic acid, ELF: Enhanced Liver Fibrosis-Test; Sens: sensitivity, Spec: specificity, PPV: positive predictive value, NPV: negative predictive value; (?): flat curve of sum of sensitivity plus specificity.

**Table 4:** Performance of non-invasive tests for diagnosis cirrhosis (F4).

Test	n	F4 prevalence in biopsy (%)	Cut-off Value	Cut off Basis	Sens (%)	Spec (%)	PPV (%)	NPV (%)	Sens+Spec (%)	Correctly classified (%)
**TE**	99	14.1 (14/99)	≥10.4 kPa	Sens + Spec = Max	100 (14/14)	89.4 (76/85)	60.9 (14/23)	100 (76/76)	189.4	90.9 (90/99)
			≥10.0 kPa	Standard [22,29]	100 (14/14)	85.9 (73/85)	53.8 (14/26)	100 (73/73)	185.9	87.9 (87/99)
			≥12.5	Standard [22,29]	85.7 (12/14)	92.9 (79/85)	66.7 (12/18)	97.5 (79/81)	178.6	91.9 (91/99)
**APRI**	104	13.5 (14/104)	≥1.1	Sens + Spec = Max	92.9 (13/14)	82.2 (74/90)	44.8 (13/29)	98.7 (74/75)	175.1	83.7 (87/104)
			≥1.0	Standard [16,29,8]	92.9 (13/14)	78.9 (71/90)	40.6 (13/32)	98.6 (71/72)	171.8	80.8 (84/104)
			>2.0	Standard [16,29,8]	42.9 (6/14)	92.2 (83/90)	46.2 (6/13)	91.2 (83/91)	135.1	85.6 (89/104)
			≥2.7 or ≥2.5	Correctly class. = Max	28.6 (4/14)	98.9 (89/90)	80 (4/5)	89.9 (89/99)	127.5	89.4 (93/104)
**FIB-4**	103	13.6 (14/103)	≥1.94	Sens + Spec = Max	100 (14/14)	74.2 (66/89)	37.8 (14/37)	100 (66/66)	174.2	77.7 (80/103)
			≥1.45(<F3)	Standard [17]	100 (14/14)	55.1 (49/89)	25.9 (14/54)	100 (49/49)	155.1	61.2 (63/103)
			>3.25(> = F3)	Standard [17]	57.1 (8/14)	93.3 (83/89)	57.1 (8/14)	93.3 (83/89)	150.4	88.3 (91/103)
**Fibro-test**	101	13.9 (14/101)	≥0.77	Sens + Spec = Max	85.7 (12/14)	75.9 (66/87)	36.4 (12/33)	97.1 (66/68)	161.6	77.2 (78/101)
			≥0.59	Standard [29,8]	100 (14/14)	55.2 (48/87)	26.4 (14/53)	100 (48/48)	155.2	61.4 (62/101)
			≥0.75	Standard [29,8]	85.7 (12/14)	72.4 (63/87)	33.3 (12/36)	96.9 (63/65)	158.1	74.3 (75/101)
**Hya**	102	13.7 (14/102)	≥76	Sens + Spec = Max	64.3 (9/14)	87.5 (77/88)	45.0 (9/20)	93.9 (77/82)	151.8	84.3 (86/102)
			>50	Standard [34]	71.4 (10/14)	68.2 (60/88)	26.3 (10/38)	93.8 (60/64)	139.6	68.6 (70/102)
			≥60	Standard [34]	71.4 (10/14)	76.1 (67/88)	32.3 (10/31)	94.4 (67/71)	147.5	75.5 (77/102)
			>110	Standard [34]	50.0 (7/14)	92.0 (81/88)	50.0 (7/14)	92.0 (81/88)	142.0	86.3 (88/102)
			≥237	Standard [36]	35.7 (5/14)	98.9 (87/88)	83.3 (5/6)	90.6 (87/96)	134.6	90.2 (92/102)
**Hepa-score**	102	13.7 (14/102)	≥0.88	Sens + Spec = Max	78.6 (11/14)	75.0 (66/88)	33.3 (11/33)	95.7 (66/69)	153.6	75.5 (77/102)
			≥0.84	Standard [19,8]	78.6 (11/14)	71.6 (63/88)	30.6 (11/36)	95.5 (63/66)	150.2	72.5 (74/102)
**ELF**	102	13.7 (14/102)	≥9.8	Sens + Spec = Max	64.3 (9/14)	88.6 (78/88)	47.4 (9/19)	94.0 (78/83)	152.9	85.3 (87/102)
			≥11.3	Standard [26]	28.6 (4/14)	98.9 (87/88)	80.0 (4/5)	89.7 (87/97)	127.5	89.2 (91/102)

TE: transient elastography; Hya: hyaluronic acid, ELF: Enhanced Liver Fibrosis-Test; Sens: sensitivity, Spec: specificity, PPV: positive predictive value, NPV: negative predictive value

#### TE (n = 99)

58 patients had TE <7.0 kPA, 15 patients 7.0 to 9.4 kPA, 8 patients 9.5 to 12.4 kPa and 18 patients > = 12.5 kPA [11,22]. TE provided an AUROC value for significant fibrosis (> = F2) of 0.85 (95% CI 0.78–0.93) and for cirrhosis (F4) of 0.97 (0.94–1.0). Optimal cut-off value for > = F2 was > = 7.2 kPa and for F4 10.4 kPa.

#### APRI-Score (n = 104)

AUROC for > = F2 was 0.76 (95% CI 0.66–0.86), for F4 0.89 (0.82–0.96). Optimal cut-off value was > = 1.1, both for > = F2 and F4. At a standard cut-off for F4 of >2.0 [23,24] 85.6% of patients were correctly classified (sensitivity 42.9%, specificity 92.2%).

#### FIB-4 Index (n = 103)

AUROC for > = F2 was 0.77 (95% CI 0.68–0.87), for F4 0.91 (0.84–0.97). Optimal cut off-value for F4 was > = 1.94. (no optimal cut off-value for > = F2, flat curve of sum of sensitivity plus specificity). At a standard cut-off for cirrhosis (> = F3) of > = 3.25 [25], 88.3% of patients were correctly classified (sensitivity 57.1%, specificity 93.3%).

#### Fibrotest® (n = 101)

AUROC for > = F2 was 0.75 (95% CI 0.65–0.85), for F4 0.84 (0.75–0.92). Optimal cut off-value for > = F2 was > = 0.6, for F4 > = 0.77. At a standard cut-off for F4 of > = 0.75 [18] 74.3% of patients were correctly classified (sensitivity 85.7%, specificity 72.4%).

#### Hyaluronic acid (n = 102)

AUROC for > = F2 was 0.70 (95% CI 0.60–0.81), for F4 0.79 (0.63–0.94). Optimal cut off-value for > = F2 was > = 60.1 μg/ml, for F4 > = 76.7 μg/ml.

#### Hepascore (n = 102)

AUROC for > = F2 was 0.68 (95% CI 0.57–0.80), for F4 0.82 (0.69–0.95). Optimal cut off-value for F4 was > = 0.88. (no optimal cut off-value for > = F2, flat curve of the sum of sensitivity plus specificity). At a standard cut-off for F4 of >0.84 [19] 72.5% of patients were correctly classified (sensitivity 78.6%, specificity 71.6%).

#### Enhanced Liver Fibrosis (ELF®)-Test (n = 102)

AUROCs for > = F2 was 0.77 (95% CI 0.67–0.86), for F4 0.82 (0.69–0.95). Optimal cut-off for > = F2 was > = 9.0, for F4 > = 9.8. At a standard cut-off for F4 of > = 11.3 [26] 89.2% of patients were correctly classified (sensitivity 28.6%, specificity 97.7%).

#### Negative predictive value of NITs

The most reliable tests to rule out significant fibrosis were: Fibroscan (9 out of 60 patients with <7.2 kPa had F2, but none F3/4 (NPV 85%)); APRI-Score (8 out of 40 patients with APRI <0.5 had F2, but none F3/4 (NPV 80%)); FIB-4 Index (10 out of 49 patients with FIB-4 <1.45 had F2, but none F3/4 (NPV 80%)); Fibrotest® (2 out of 20 patients with Fibrotest® <0.32 had F2, but none F3/4 (NPV 90%)); and ELF®-Test (out of 13 patients with ELF® <7.7 none had > = F2 (NPV 100%)). Among 71 patients with hyaluronic acid <60ug/ml and 38 patients with Hepascore <0.5, there were patients not only with F2, but also with severe fibrosis (F3) and even cirrhosis (F4) (NPV (<F2) 76% for both tests).

#### Combination of different tests

Different algorithms combining TE and serum biomarkers have been proposed [27,28]. According to our data the combination of TE and blood marker does not increase the diagnostic accuracy for cirrhosis. Using a standard cut-off of TE > = 9.5 kPa to detect severe fibrosis (> = F3) as an indication for HCV-treatment, one fourth of baseline biopsies (26 of 105) could have been avoided. However, with this approach one participant with severe fibrosis (F3) in biopsy would not have received treatment and six participants with mild fibrosis (F1) would have been treated without urgent need. The combination of TE and serum biomarkers was safer in ruling out significant fibrosis (F2) than TE alone. If TE was <7.0 kPa and FIB-4 <1.45, the chance of absence of significant fibrosis was high and liver biopsy could have been deferred too. With this approach 36 additional baseline biopsies (34%) could have been saved, but would have missed 5 patients with F2-fibrosis. However, no patient with liver stiffness <7.0 kPa or FIB-4 Index <1.45 had F3 or F4 fibrosis in liver biopsy.

### Longitudinal follow-up

All participants with a follow up of at least 1 year (n = 101) were on ART (86 at baseline, 15 started during follow up). ART in this group usually included two nucleos(t)ide reverse transcriptase inhibitors (NRTIs: Lamivudine or Emtricitabin (n = 80) and Tenofovir (n = 52) or Abacavir (n = 40) and a protease inhibitors (predominantly boosted Lopinavir (n = 40) or boosted Atazanavir (n = 29)). 20 patients were treated with a combination therapy including a non-nucleoside reverse transcriptase inhibitor (NNRTI: Efavirenz (n = 11), Nevirapine (n = 66), Etravirine (n = 3)). 87 (83%) of the 105 enrolled participants had a complete follow up of 3 years.

Eight patients died during the follow-up period (1 liver failure, 1 hepatocellular carcinoma, 1 suicide, 1 bronchial neoplasm, 1 pneumonia, 1 HIV-associated, and 2 from an unknown cause but probably related to substance abuse). Nine patients dropped out due to several reasons (e.g. moving away, non-adherence). One patient was excluded from the analysis because of liver transplantation (due to cholangiocarcinoma) during follow up. One patient was hospitalised due to a flare of hepatitis. 20 patients (17 with complete follow up) received treatment of HCV infection (all patients with peg-interferon and ribavirin, one patient in combination with faldaprevir). 46% (9/20 respectively 8/17) had a sustained virologic response 24 weeks after end of treatment (SVR24).

#### Fibrosis assessment during follow up period

87 (83%) of 105 participants with a complete follow-up of 3 years were analysed. According to the results of the baseline evaluation, after 3 years, the biochemical analysis was restricted to 3 indirect serum-markers (APRI-Score, FIB-4 Index, and Fibrotest).

#### Change of non-invasive tests over 3 years

**Fig 5:**
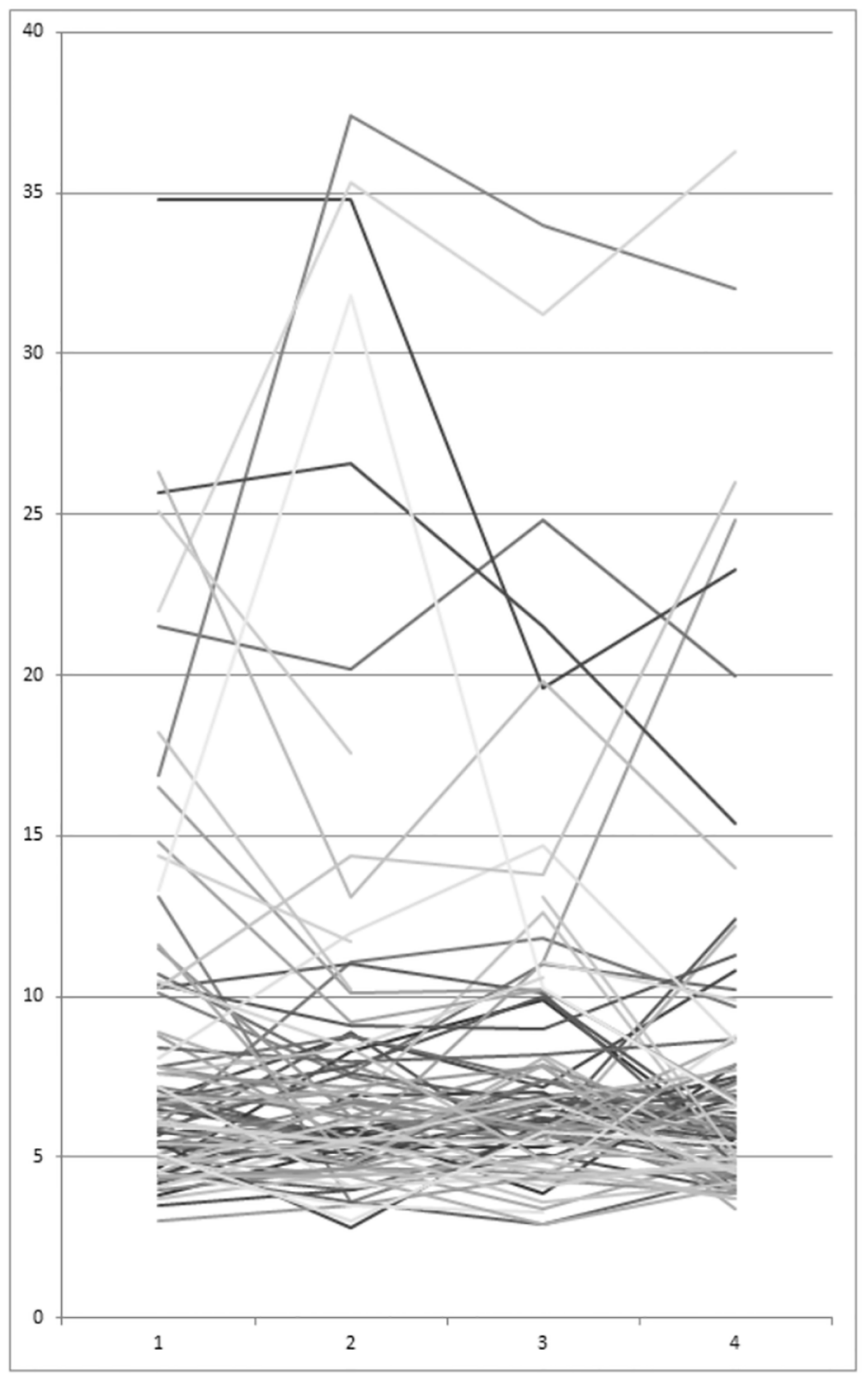
Change in transient elastography values (kPa) over time (3 years).

Change in TE is shown in Fig 5. Mean TE (n = 82) was 8.2 kPA (range 3.0–34.8) at baseline and 7.9 kPA (range 3.4–36.3) after 3 years. Mean APRI-Score (n = 85) was 0.85 (range 0.10–4.16) at baseline and 0.81 (range 0.05–5.77) after 3 years. Mean FIB-4-Score (n = 85) was 1.74 (range 0.24–7.40) at baseline and 1.95 (range 0.27–7.73) after 3 years. Mean Fibrotest (n = 85) was 0.56 (range 0.04–0.97) at baseline and 0.55 (range 0.04–0.97) after 3 years. Liver-stiffness, APRI and Fibrotest did not significantly change between baseline and follow-up examination after 3 years. There was weak evidence (p = 0.07) for an increase of FIB-4 (mean difference: 0.22 (95% CI: -0.02 to 0.45)).

#### Impact of HCV-treatment and treatment outcome on results

Mean TE was 8.1 kPA at baseline and 8.0 kPA after 3 years in patients without HCV-treatment (n = 66), mean TE declined from 8.5 kPa to 6.1 kPa in patients with SVR (n = 8) and from 9.9 kPa to 9.0 kPa in patients with HCV-treatment but without SVR (n = 8).

#### Change of histopathological stage of liver fibrosis

Half of patients with complete follow up refused a second liver biopsy. On follow up biopsy (n = 42) no or mild fibrosis (METAVIR stage F0 or F1) was observed in 26 patients. 10 patients had moderate (F2), 1 severe fibrosis (F3) and 5 cirrhosis (F4). Among 38 patients with the potential to increase in fibrosis stage (F0-F3 at baseline) 10 were progressors (8x 1-stage, 1x 2-stage (F0 to F2) and 1x 3-stage (F1 to F4) increase of fibrosis over 3-years), and 28 patients were non-progressors (27 stable, 1 regression of fibrosis). Only 5 patients with HCV-treatment had paired liver biopsy (4 stable, 1 regression of fibrosis).

#### Non-invasive tests in patients with paired liver biopsy and prediction of fibrosis progression

Among the 38 participants with a complete data set (paired liver biopsy and NITs available at baseline and after three years), mean increase in TE was significantly higher in progressors (+3.35 kPA) than in non-progressors, (-0.15 kPA, p = 0.01). Per kPa increase over 3 years the odds of fibrosis progression was 1.3fold increased (95% CI: 1.01–1.80, p = 0.045). (APRI: progressors: +0.36, non-progressors: -0.30, p = 0.03; OR per unit increase: 5.9 (0.85–42), p = 0.073; FIB-4: progressors: +0.75, non-progressors:- 0.06, p = 0.03; OR per unit increase: 3.2 (0.84–11.9), p = 0.088; Fibrotest: progressors: +0.04, non-progressors: -0.03, p = 0.269)

Rapid liver disease progression (increase of >1 Metavir fibrosis stage during 3 years) was observed in 2 participants (A: F0 to F2 (2 stages) and B: F1 to F4 (3 stages)). Both patients would have been detected by regular follow-up of TE at yearly intervals (A: steady increase of liver stiffness over time from 5.4 to 7.5 kPa; B: heterogenic liver fibrosis suspected at baseline, increase from <7.0 to 12.6 kPa within 2 years). FIB-4 Index was also helpful (A: 4.51 at the end of the study; B: above <1.45 (standard cut-off for excluding significant fibrosis) after one year of follow-up).

## Discussion

In this prospective cohort study of 105 HIV/HCV co-infected participants, we first compared the diagnostic performance of non-invasive tests versus liver biopsy, and second, we determined progression of liver disease during a three year follow-up. First, we found that TE was the non-invasive test with the highest diagnostic accuracy in detecting both significant liver fibrosis (METAVIR > = F2) and liver cirrhosis (METAVIR F4) compared to liver biopsy as the gold standard. Three indirect serum markers (APRI-Score, FIB-4 Index, Fibrotest) and one direct serum marker (ELF-Test) were secondary alternatives, whereas performance of the other two direct serum markers (hyaluronic acid and Hepascore) was worse. Routinely available tests (APRI, FIB-4) performed as good as the patent-registered and more expensive blood tests (Fibrotest and ELF). Second, non-invasive tests did not change significantly during the 3-year follow-up, suggesting slow liver disease progression in a majority of HIV/HCV co-infected patients on ART. However, one in four participants with paired liver biopsy showed progression by 1 METAVIR fibrosis stage and two patients (5%) were rapid progressors (progression by >1 stage).

The diagnostic accuracy of all NITs rose with increasing stage of fibrosis (Figs 1–4), as described in the review of Chou and Wasson [7]. The accuracy of TE in predicting significant fibrosis and cirrhosis was clearly better than that of the six tested blood biomarkers. This finding is in concordance with the results of Degos et al. who compared TE, Fibrotest, Fibrometer, APRI-Score, and Hepascore in 1307 patients with viral hepatitis in France [29]. Performance of TE in our study was even better than in the mentioned analysis (AUROC for > = F2 0.85 vs 0.76; for F4 0.97 vs 0.90). Our results also correspond to the findings of Castera et al. who analysed the diagnostic accuracy of TE, Fibrotest, APRI and two algorithms combining TE and Fibrotest (Castera) or APRI and Fibrostest (SAFE) in 116 HIV/HCV co-infected patients [30]. Castera found that TE and Fibrotest had a similar diagnostic accuracy for significant fibrosis (AUROC = 0.87 and 0.85 respectively), whereas for cirrhosis TE had the best accuracy (AUROC = 0.92). AUROC for TE was comparable in our study for F> = 2 and even higher (0.97) for cirrhosis (F4). Thus, the use of combining different NITs does not seem to improve diagnostic performance. In contrast to our results, NITs using markers such as α-2-macroglobulin or apolipoprotein A1 (Fibrotest or Hepascore) tended to perform slightly better than APRI-Score and FIB-4 Index in other studies [9,8]. Both tests include total bilirubin (Table 1). Thus, Atazanavir-based ART (21 study participants concerned), which can cause elevated total bilirubin, may result in higher values and false positive results (i.e. overestimation of fibrosis stage). On the other hand HIV-induced thrombocytopenia could lead to an overestimation of liver fibrosis by FIB-4 Index and APRI-Score [31]. Sebastini et al. investigated the effect of aetiology of chronic liver disease (CLD) on the performance of different fibrosis biomarkers in a large cohort of 2411 patients with CLD [32]. APRI and Fibrotest exhibited the best performance (AUROCs of 0.7 for > = F2 and F4) and the accuracy of these NITs was not significantly inferior in HIV/HCV co-infection (n = 158) compared to HCV mono-infection (n = 1810). In our HCV/HIV co-infected cohort with most participants on ART, FIB-4 and APRI performed as good as or even better than the more sophisticated and expensive composite markers. Hyaluronic acid, a NIT not likely affected by HIV-infection or ART, was less reliable in correctly detecting F2 and F4-fibrosis (AUROCs 0.70 and 0.79) in our study. Hepascore (which also contains hyaluronic acid, but also total bilirubin, GGT, α-2-macroglobulin, age and sex) did not perform better than hyaluronic acid alone.

In contrast to sensitivity and specificity, positive and negative predictive values depend on prevalence. With increasing prevalence, the positive predictive value (PPV) increases, whereas the negative predictive value (NPV) decreases. Accordingly, in our setting with a > = F2 prevalence of 36%, a F4 prevalence of 13%, and a single optimal cut-off (where the sum of sensitivity and specificity was maximal), absence of cirrhosis (F4) could be predicted with NPVs of 94–100%, whereas the prediction of absence of significant fibrosis (<F2) was worse (NPVs 74–85%). On the other hand, PPVs for the presence of significant fibrosis (> = F2) were generally higher than those for the presence of cirrhosis (F4) (55–79% versus 33–61%; Tables 3 and 4). Higher cut-off values can be used to increase specificity and PPV. Lower cut-off values improve sensitivity and NPV. For some of the non-invasive tests, there is a lower standard cut-off value for the absence and a higher standard cut-off value for the presence of significant fibrosis and cirrhosis, respectively (Table 2).

The strength of our study is the longitudinal follow-up of patients. Vermehren et al. Assessed liver fibrosis, associated risk factors and fibrosis progression in 202 consecutive HIV-infected individuals (35 with HCV co-infection) using TE (and Fibrotest at baseline) [33]. HCV co-infection was the predominant factor associated with prevalence of significant fibrosis. Up to 45.5% of HIV mono-infected patients had chronic elevation of liver enzymes. However there was no evidence for fibrosis progression (assessed by TE) during a median follow-up of two years in 68 HIV mono-infected individuals. Because of small numbers no statement on fibrosis progression in HIV/HCV co-infected patients was possible. Our study had a follow-up of 3-years, 83% of participants had a complete follow-up. However, only 40% of the participants agreed to have a second liver biopsy which shows a higher acceptance for non-invasive procedures. Since only four participants had severe fibrosis (F3) at baseline, we restricted our validation of the non-invasive tests to the detection of significant fibrosis (> = F2) and cirrhosis (F4). Another limitation of our study is the lack of an optimal cut-off value for APRI, FIB-4, and Hepascore for the detection of > = F2. We aimed at the definition of an optimal cut-off value both for the presence and absence of significant fibrosis using a cut-off value with a maximized total for sensitivity and specificity. For APRI, the optimal cut-off value was identical for ≥F2 and F4 (> = 1.1 in both cases). We recommend the use of standard cut-off values (<0.5 for the absence and >1.5 for the presence of significant fibrosis; <1.0 for the absence and >2.0 for the presence of cirrhosis), which also show some overlap and proved reliable in our setting (Tables 2–4). For FIB-4 and Hepascore, the curve of the sum of sensitivity and specificity had a plateau without distinct maximum. The highly questionable optimal cut-off values for > = F2 were higher than that for F4. For the absence of significant fibrosis, FIB-4 <1.45 can be used (NPV 80%) (actually a cut-off to rule out > = F3). For Hepascore, there is a single standard cut off-value for absence/presence of significant fibrosis at 0.5 (NPV 76%, PPV 42%).

Our results support the new recommendations by EASL [31] to use non-invasive tests (NITs) as first line tests to stage liver fibrosis, also in HIV/HCV co-infection. According to our findings and these new guidelines, liver biopsy is only needed if repeated non-invasive tests show discordance. Transient elastography (TE) is the NIT of choice to stage liver fibrosis in HIV/HCV co-infected patients. Blood markers (APRI-Score, FIB-4 Index, Fibrotest, and ELF-Test) were less reliable alternatives and should be used only if TE is not available or impossible to perform. In this situation our data support the use of a simple, low-cost blood score (FIB-4 or APRI) rather than complex composite markers. Non-invasive tests of liver disease did not change significantly during a follow up of three years, suggesting slow liver disease progression in a majority of HIV/HCV co-infected patients on ART. Fibrosis progression however can be rapid in a minority of patients. Longitudinal follow-up at yearly intervals with assessment of liver fibrosis by NITs, preferably TE, is a reasonable approach in HIV/HCV co-infection if HCV-treatment is deferred.

## Supporting Information

S1 Dataset Subject characteristics, blood values, transient elastography values and histopathological results at participant-level. (XLS)
